# Fear of abortion and emotional divorce in women with minor thalassemia: a population-based study in Yazd, Iran

**DOI:** 10.1186/s12905-021-01551-7

**Published:** 2021-12-07

**Authors:** Zohreh Rahaei, Mohammad Ali Sahami, Reza Bidaki

**Affiliations:** 1 Department of Health Education and Health Promotion, School of Health, Shahid Sadoughi University of Medical Sciences, Yazd, Iran; 2 Department of Psychiatry, Research Center of Addiction and Behavioral Sciences and Diabetes Research Center, Shahid Sadoughi University of Medical Sciences, Yazd, Iran

**Keywords:** Abortion, Fear, Emotional, Divorce, Minor Thalassemia, Women

## Abstract

**Background:**

Thalassemia is the most common genetic disorder in humans that can be controlled and treated by, premarital screening, prenatal diagnosis and abortion. Aortion can be a critical issue for many families based on the cultural and religious backgrounds and have different consequences for couples, such as emotional divorce. Therefore, the present study aimed to investigate the association between fear of abortion and emotional divorce in women with minor thalassemia in Yazd City, Iran.

**Materials and methods:**

This retrospective study was conducted on 61 women with minor thalassemia (case group) and 100 healthy women (control group), who referred to health centers in Yazd. The census sampling was applied to select the case group and multistage (cluster andsimple) random sampling was adopted to select the control group. Data were collected using Gutman Emotional Divorce Questionnaire and a researcher made scale for measuring fear of Abortion. Data were analyzed by SPSS using descriptive statistics and chi-square, independent *t*-test, Pearson correlation, and linear regression.

**Results:**

The mean scores of emotional divorce (6.62 vs. 4.26) and fear of abortion (25.85 vs. 17.4) were higher in the case than control group (*P *˂ 0.01). There was a positive and significant correlation between fear of abortion and emotional divorce in the case (*P *˂ 0.05, r = 0.275) and control (*P *˂ 0.05, r = 0.570) groups. Fear of abortion in the case group predicted 25% of the variance in emotional divorce.

**Conclusion:**

Given the high level of fear of abortion in women with minor thalassemia and its effect on increasing the emotional divorce, designing and implementing psychological interventions with ongoing follow-up are recommended for thalassemia carrier couples.

## Introduction

Thalassemia is the most common genetic disorder in humans. In patients with thalassemia, the body does not produce enough hemoglobin, which is an important part of red blood cells. Approximately, 150 million people carry the beta thalassemia gene throughout the world [1]. The disease is most prevalent in the Mediterranean regions, parts of North and West Africa, the Middle East, the Indian subcontinent, the Far East, and Southeast Asia [2, 3].

Iran, like many countries in the region, has a relatively large number of patients with thalassemia. Beta thalassemia major is the most common type of thalassemia in Iran. More than 30 thousand people are affected and about 2–3 million people carry the disease in Iran [4, 5].

Thalassemia can be controlled and treated by screening, genetic counseling, prenatal diagnosing and terminatingon of pregnancy [6, 7]. Although the public level of awareness has been increased (to a good level), the prevalence of marriage between thalassemia carriers shows that the success rate is only 20% in this field and most thalassemia carrier couples marry each other. This is a matter of concern and more measures should be taken to influence the individuals' attitudes. Individuals with thalassemia should have high levels of awareness, so that they can behave appropriately [8–10]. Moreover, most carrier studies and measures were focused on children with thalassemia major and their problems. In other words, thalassemia carrier couples have not been considered in the case of having no child with beta thalassemia [4, 6, 11].

Termination of pregnancy is one of the most important measures in preventing the birth of infants with thalassemia major. The prenatal diagnostic test results show that the fetus has thalassemia major, abortion is performed in accordance with legal and religious issues. However, abortion can be a critical issue for many families based on the cultural and religious backgrounds [12].

A review study found that women with a history of abortion were more likely to experience psychological trauma such as depression and anxiety [13]. The psychological and physical problems resulting from abortion can reduce the quality of life by affecting different aspects of women's life and by creating physical and marital problems. Grief, as one of the major risk factors for mental illness, sadness, depression, and anxiety, occurs in over 30% of women who had abortions. In 5–10% of cases, the severity and persistence of these symptoms lead to major depression [14, 15]. Steinberg and Biggs believe that, factors such as mental health status before abortion, domestic violence, desire to pregnancy, economic status, etc. are effective in the development of depression and anxiety after abortion [16, 17]. Other researchers believe that abortion is a stressful factor that causes psychological complications [18].

However, psychological complications may occur after abortion due to any reason. According to Lok, the psychological symptoms of women with a history of abortion are similar to the mental states of mothers who have lost their babies [19]. Given the fact that the prevalence and severity of the maternal and fetal interest vary from person to person, some mothers feel interest to the fetus as soon as they become aware of their pregnancy and are afraid of having an abortion. Terminating the pregnancy can be a painful decision with consequences [20, 21]. According to a study in Iran, all mothers were afraid of having a legal abortion and 35% of the participants expressed their fears by crying [22].

Pregnancy and fear of abortion in women with thalassemia cause a Marital Conflicts and emotional reactions, which in turns may lead to emotional divorce [23].

Emotional divorce, also called silent divorce, is a very important type of divorce, which is not recorded anywhere. Emotional or latent divorce is almost twice more common than formal divorce [23]. In emotional divorce, the woman seeks isolation, does not consider herself a wife, and continues to live only because of social, family, and cultural conditions. The dimensions of conflict can be classified into physical, verbal, psychological, and sexual challenges [24]. Emotional divorce symbolizes the existence of a problem in a healthy and mature relationship between couples. This small-scale communication challenge can turn into a complicated problem and disrupt human relationship. As a result, the foundation of the family is shaken, the moral and social foundations of the whole social system are shaken, and the society is directed to different problems [25].

Few studies investigated couples with thalassemia since these people do not have any specific physical problems. Furthermore, couples with thalassemia are faced with many psychological and emotional problems, especially women who should face pregnancy and deal with fear of abortion [22]. In addition, a large number of thalassemia carrier couples live in Yazd, who got married despite all the warnings and educations maintaining that two carriers should not marry with each other. Consequently, the present study aimed to investigate the association between fear of abortion and emotional divorce among women with minor thalassemia in Yazd City, Iran. Later, the secondary prevention measures were also addressed.

## Materials and methods

This retrospectivestudy was conducted among all thalassemia carrier couples (n = 80) identified using the census sampling in Yazd City until 2018. These couples had undergone premarital carrier screening, but married despite the counseling. These couples are regularly followed up by health centers using timely and free preventive interventions.

Among these couples, women were asked to complete the questionnaires. Since 19 women were not willing to participate in the study, the questionnaires were collected from 61 participants.

The control group were randomly selected from the clients of comprehensive health service centers. Four centers were selected from all comprehensive health service centers in Yazd (n = 22 centers) using cluster sampling. Later, 25 women were selected using simple random sampling from each center. The control group were matched in terms of age with the case group.

The following tools were used in this study.

The demographic information questionnaire that included participants' age, duration of marriage, number of children, level of education, occupation, and number of abortions.

The Emotional Divorce Questionnaire, designed by John Guttman (2008), includes 24 items about various aspects of life that one may agree with or disagree with. In other words, the items should be answered using 'yes' or 'no' options. The 'yes' responses receive one score and the 'no' answers receive no score. The higher number of 'yes' responses shows higher probability of emotional divorce. The validity of this questionnaire was examined and confirmed by a panel of experts and its reliability was confirmed using Cronbach's alpha coefficient of 0.8.

Fear of abortion scale: This researcher-made scale consisted of 9 questions designed using previous similar studies. The questions should be answered on a 5-point Lickert scale using Never (no score), Almost (1 score), Relatively (2 scores), Highly (3 scores), and Very highly (4 scores) options. The validity of this questionnaire was examined and confirmed by a panel of experts and its reliability was confirmed using Cronbach's alpha coefficient of 0.83.

### Data analysis

In order to analyze the data, SPSS statistical program was used. Data were analyzed using descriptive (frequency and mean) as well as inferential statistics by running Chi-square, independent t-test, Pearson correlation, and linear regression.

### Ethical considerations

This study was approved by the Research Ethics Committee of the Health School, Yazd Shahid Sadoughi University of Medical Sciences with the code IR.SSU.SPH.REC.1396.126. The participants were provided with the necessary explanations about the study purpose and method prior to the research. Women who were willing to participate in the study were asked to sign written informed consent forms. Moreover, questionnaires were coded to keep information confidential.

## Results

Most participants in the case and control groups had high school (39%) and bachelor's degree or higher (46.9%), respectively. The majority of women in both groups were housekeepers. The two groups were not similar in terms of demographic variables (Table 1).

**Table 1:** Frequency distribution of demographic variables in the case and control groups

Variable	Categories	Case group		Control group		*P* value
		Number	Percentage	Number	Percentage	
Educational level	Primary school	8	13.6	2	2	0.02
	Secondary school	9	15.3	16	16.3	
	High school	23	39	34	34.7	
	Bachelor’s degree and higher	19	32.2	46	46.9	
Number of children	0	9	14.8	0	0	< 0.001
	1	29	47.5	15	15.2	
	2	15	24.6	77	77.8	
	3	8	13.1	7	7.1	
Occupation	Housekeeper	42	70	83	83.8	0.039
	Employee	18	30	16	16.2	

The mean score of emotional divorce and fear of abortion was higher in the case group than the control group (Table 2).

**Table 2:** Distribution of mean and standard deviation of fear of abortion and emotional divorce in the case and control groups

Variable	Case group		Control group		Range of scores	*P* value
	Mean	Standard deviation	Mean	Standard deviation		
Emotional divorce	6.62	4.34	4.26	4.72	0–24	0.001
Fear of abortion	25.85	80.6	17.40	6.26	0–36	0.010

A positive correlation was observed between the number of abortions and emotional divorce in the case group (*P* < 0.05, r = 0.275) as well as between the number of abortions and fear of abortion in the control group (*P* < 0.05, r = 0.570).

Apositive correlation was found between fear of abortion and emotional divorce in the case (*P* < 0.01, r = 0.504) and control (*P* < 0.05, r = 0.241) groups.

The fear of abortion predicted 25% and 5% of the variance of emotional divorce in the case and control groups, respectively (Table 3). Women in the control group were selected randomly and differed from the case group in terms of all demographic variables except for age. So, the effects of demographic variables were controlled to ensure the relationship between fear of abortion and emotional divorce in the regression equation. Only fear of abortion had a significant effect in predicting emotional (Table 4).

**Table 3:** Regression analysis of fear of abortion as a predictor of emotional divorce in the case and control groups

	Independent variables	Standardized beta	*P* value	R^2^	Dependent variable
Fear of abortion	Case group	0.276	0.001	0.25	Emotional divorce
	Control group	0.192	0.029	0.05

**Table 4:** Regression analysis of covariates possibly associated with emotional divorce in women with thalassemia

Independent variables	Standardized Beta	*P* value	R^2^	Dependent variable
Fear of abortion	0.530	0.001	0.35	Emotional divorce
Age	− 0.237	0.399		
Length of marriage	0.141	0.643		
Number of children	0.211	0.335		
Educational level	− 0.044	0.831		
Income	0.140	0.437		
Number of abortion	0.258	0.074		

## Discussion

Thalassemia is the most common genetic disorder in humans, so that approximately 150 million people carry the beta-thalassemia gene worldwide [1]. Although Termination of pregnancy is used as a successful method [26], it has unavoidable consequences for couples such as the occurrence of 'emotional divorce'. Couples with thalassemia, especially women, are faced with different psychological and emotional consequences and problems due to pregnancy and fear of abortion [22]. So, the present study aimed to investigate the relationship between fear of abortion and emotional divorce among thalassemia carriers in Yazd.

The case and control groups were significantly different in terms of all demographic variables, except for age that was matched prior to the study. To control the effect of demographic variables on emotional divorce, linear regression analysis was used. The results showed that none of the demographic variables was significantly associated with emotional divorce.

The mean scores of fear of abortion and emotional divorce were higher in the case group than the control group. As some studies mentioned, abortion is a stressful and inappropriate measure that causes psychological problems in the family. So, most pregnant women avoid induction of abortion even legally [27–29]. Similar studies on thalassemia-carrying couples noted various emotional symptoms such as anxiety, hopelessness, and shock in 50% of cases and negative psychological symptoms such as grief and depression in 71% of families [30, 31]. In a study, all mothers with thalassemia feared having a legal abortion and 35% expressed their fear by crying [22]. Palomba reported that women with a history of abortion were very dissatisfied with this condition. Considering the modern treatments of thalassemia, Klanago et al. believed that some families preferred to give birth to thalassemia children over an abortion [32, 33].

The results of a research by Catherine et al. (2010) showed that lack of pre-abortion counseling led to post-abortion psychological problems [34]. This result indicates that counseling can be suggested as an interventional efficient therapy in reducing the fear of abortion among pregnant or non-pregnant women with thalassemia. In such cases, the only optionssolution is to provide supportive care. In other words, interventions to prevent psychological consequences and emotional divorce are recommended in cases where thalassemia couples intend to have children.

Abortion in healthy women can also occur due to various reasons and is not a pleasant event in any case, but it should be borne in mind that abortion occurs quite suddenly and unexpectedly in healthy women and its complications are more focused on the post-abortion consequences. However, the fear of abortion accompanies women with thalassemia from the very beginning of pregnancy. Higher levels of fear in these women can cause psychological problems with regard to pregnancy. This also can affect women's sexual and emotional relationships with husbands, which will lead to emotional divorce in long term.

Emotional divorce was more frequent in women with thalassemia who had experienced more abortions. The fear of abortion was not associated with the number of abortions in women with thalassemia. In other words, all female carriers of thalassemia were afraid of abortion. In healthy women, only increased number of abortions increased the fear of abortion, which had no effect on incidence of emotional divorce in this group, since abortions were due to causes other than the couple's illness.

There was a positive association between fear of abortion and emotional divorce in the case and control groups. Fear of abortion predicted 25% of the variance of emotional divorce in the case group. In a research, couples reported nervous tension, fear, and anxiety about having a child with thalassemia and its consequences. They were discouraged, shocked, and despaired hearing the news that their child had thalassemia. The couples believed that diagnosis of thalassemia in their child affected the family's mental state and marital relationships greatly, reduced exhilaration of the family, and decreased affection between couples. In some families, the women underwent a tubectomy to avoid giving birth to another baby with thalassemia. In some other cases, family disputes escalated due to emotional divorce and led to divorce [35]. As a result, the breakdown of the couple's relationship or emotional divorce is an unfortunate consequence of pregnancy and fear of abortion in women with thalassemia.

## Conclusion

Given the high level of fear of abortion in women with thalassemia and its adverse effects on emotional divorce, holding counseling sessions for couples with thalassemia can be effective in preparing them for accepting an abortion and managing their stress. These educational courses should provide different methods to treat mental and psychological health problems of women with thalassemia, especially fear. Thalassemia patients and their families, especially husbands of women with thalassemia, should be provided with supportive and counseling services.

Furthermore, thalassemia patients on the verge of marriage should be provided with the findings of this research about consequences of their marriage. They should be prepared to face these consequences and take timely measures to receive psychological interventions and supportive services in this regard.

## Limitations

This study was limited to women living in Yazd, future researchers are required to conduct similar studies among larger statistical communities. In addition, questionnaires were administered as the main data collection instrument in this study. Although collecting data through questionnaires is common in most studies, we suggest other researchers to collect the required data using several different methods.

## Data Availability

The datasets used and/or analysed during the current study are available from the corresponding author on reasonable request.
